# Patients’ Voices and the Assessment of Medical Trainees: A Discourse Analysis

**DOI:** 10.5334/pme.2588

**Published:** 2026-07-06

**Authors:** Christina St-Onge, Isabelle Boulais, Linda Bergeron, Vincent Dion, Iris Le Sieur, Mélanie Marceau, Marie-Eve Poitras, Tim Dubé, Daniel J. Schumacher

**Affiliations:** 1 Department of Medicine, Université de Sherbrooke, Sherbrooke, Québec, Canada; 2 Faculty of Medicine and Health Sciences, Université de Sherbrooke, Sherbrooke, Québec, Canada; 3 School of Nursing, Université de Sherbrooke, Sherbrooke, Québec, Canada; 4 Department of family medicine and medical emergency at Université de Sherbrooke, Sherbrooke, Québec, Canada; 5 Department of family medicine and emergency medicine, Université de Sherbrooke, Sherbrooke, Québec, Canada; 6 Holder of the Chaire de recherche en pédagogie médicale Paul Grand’Maison de la Sociétédes médecins de l’Universitéde Sherbrooke, Université de Sherbrooke, Sherbrooke, Québec, Canada; 7 Department of Pediatrics, Cincinnati Children’s Hospital Medical Center/University of Cincinnati College of Medicine, Cincinnati, Ohio, United States

## Abstract

**Introduction:**

Researchers have emphasized the need to integrate patients into medical education, specifically advocating for patient-based assessments within medical curricula. However, there is a critical gap in understanding how patient involvement is conceptualized and operationalized within assessment practices. Thus, we aimed to gain a deeper understanding of the current discourses surrounding the integration of real patients in the assessment of medical trainees.

**Methods:**

We performed a Discourse Analysis using Hodges’ empirical discourse methodology. Coding of the 54 included articles initially focused on “conceptualization,” “ways patients participate,” “opportunities created,” “challenges,” and detailed aspects of “assessment,” including content, development, validation, and format. The team iteratively discussed the codes and themes until they reached a consensus about the data interpretation.

**Results:**

We identified three distinct—yet not always mutually exclusive—discourses: (1) patients as survey-fillers, (2) patients as feedback providers, and (3) patients as part of programmatic assessment.

**Discussion:**

By illuminating these discourses, this study deepens our understanding of why patient involvement takes the forms it does and suggests that moving toward positioning patients as part of programmatic assessment may offer a more meaningful basis for patient partnership in the assessment of medical trainees.

## Introduction

Despite a longstanding call for a patient-centered approach in healthcare and medical education, the concrete integration of real patients into the assessment of medical trainees remains surprisingly under-explored [1]. While much of the existing published research has been about quantifying what patients assess and tallying the assessments needed for reliable representations of trainees’ performance, there is a critical gap in understanding how patient involvement is conceptualized and operationalized within assessment practices. In other words, we have not yet identified the different narratives and assumptions that could shape practices, policies, and experiences about the integration of real patients in the assessment of medical trainees. By not doing so, we may unwillingly perpetuate superficial integration and limit the potential richness that real patient assessment data could add by providing a more holistic representation of perspectives [1].

While Standardized Patients (SPs) have been a part of the assessment landscape for some time, the engagement of real patients—who bring authentic lived experience—is far less developed and often confined to satisfaction surveys driven by resource and psychometric concerns [1,2,3,4,5,6]. SPs have contributed to the assessment of medical trainees for several decades [7,8,9,10,11]. In this context, they have predominantly been asked to assess “soft-skills” (i.e., communication and interpersonal skills, and professionalism) [1,2]. Authors often justify that patients can ‘only’ assess ‘soft-skills’ based on lack of correlation between patients and physicians scores when assessing content such as medical expertise (e.g., Castonguay et al.; Reinders et al.; Brinkman et al.) [12,13,14]. Similarly, when integrated, real patients (RP) are predominantly asked about their satisfaction [1]. Decisions about satisfaction-based patient assessments seem informed mainly by how many forms are required to achieve reliability [2] and what resources are required to achieve that level of reliability [2]. These patterns reveal a persistent instrumental view of patients in assessment—valued for their reliability or convenience rather than for the depth of their experiential knowledge.

We posit that implicit assumptions and underlying power relations influence how real patients are integrated into the assessment of medical trainees and warrant closer examination to inform future practice. Khalife et al. [2] and Nemir et al. [1] mapped the breadth of patient-based assessment across contexts. By doing so, they documented contexts in which patients provided assessment of medical trainees, barriers and facilitators in doing so, the frequency of it happening, etc. Building on their findings, we aim to gain a deeper understanding of the current discourses surrounding the integration of real patients in the assessment of medical trainees. Through Discourse Analysis, we move beyond descriptive mapping of the current literature on patient-cased assessment to analyze the discourses that shape, legitimize, and sustain particular forms of patient involvement in assessment.

## Methods

Our study draws on discourse theory as proposed by Mills [15], and utilizes Discourse Analysis as a methodology. Discourse Analysis is particularly suited to this aim, as it allows us to uncover how language constructs and legitimizes particular understandings of patient involvement, thereby revealing the normative and power-laden assumptions embedded in assessment practices. By examining the discursive patterns within the literature, we can trace how certain ways of representing patients become dominant while others are marginalized. Specifically, we employed an approach that Hodges et al. [16] have classified as ‘empirical discourse analysis.’ In doing so, we examined how the literature constructs and frames the integration of patients into the assessment of medical trainees, with particular attention to the language used to normalize, justify, or contest particular forms of patient involvement.

### Identifying and selecting the relevant literature

Our goal was to compile a sufficient number of relevant manuscripts addressing the topic of interest, specifically the integration of real patients into the assessment of medical trainees. Since our objective was not to create an exhaustive archive, as would be expected in a scoping or systematic review, we utilized a fluid and iterative approach to building the pool of articles utilized in this study, rather than a traditional systematic method. This enabled us to continue adding to the archive until we reached a point of data sufficiency/information power. More specifically, we aimed to include manuscripts as long as there was “nothing new” extracted from the manuscripts [17,18].

First, the team members identified key articles that they used as seminal references in the Spring of 2022 on the topic of integrating real patients into the assessment of medical trainees. The team members aimed to identify papers that specifically addressed this topic without necessarily using known or generic keywords. We also aimed to garner different points of view and types of articles, such as commentaries and knowledge synthesis, to avoid only accessing psychometric studies about assessment forms that would offer a limited perspective. In other words, by starting the data collection using a pool of articles, we wanted to avoid missing papers that would not have use traditional keywords or MeSH terms. The team members’ experience and expertise are varied, and allowed the initial pool of articles to reflect that. Key references were not time-bound, but were considered a good overview of the topic of interest. The first author (CSTO) coded all six key articles (Table 1), and identified 140 potential snowball references.

**Table 1:** List of Key Articles.


El-Haddad C, Damodaran A, McNeil HP, Hu W. A patient-centered approach to developing entrustable professional activities. Acad Med. 2017;92(6):800–8. *An empirical article on the topic of interest, with a comprehensive list of references. Khalife R, Gupta M, Gonsalves C, Park YS, Riddle J, Tekian A, Horsley T. Patient involvement in assessment of postgraduate medical learners: a scoping review. Med Educ. 2022;56(6):602–13. *A recently published knowledge synthesis on the topic that offered a great stepping stone to explore the literature with a different question. Malau-Aduli BS. Patient involvement in assessment: how useful is it? Med Educ. 2022;56(6):590. *A recent empirical article on the topic published in a top tier journal. Moreau K, Eady K, Jabbour M. Patient involvement in resident assessment within the Competence by Design context: a mixed-methods study. Can Med Educ J. 2019;10(1):e84–102. *An empirical article on the topic of interest, with a comprehensive list of references. Sebok-Syer SS, Gingerich A, Holmboe ES, Lingard L, Turner DA, Schumacher DJ. Distant and hidden figures: foregrounding patients in the development, content, and implementation of entrustable professional activities. Acad Med. 2021;96(7 Suppl):S76–80. *A position paper that explored and proposed new ways of thinking about the integration of real patients in the assessment of trainees. Ten Cate O. Entrustment decisions: bringing the patient into the assessment equation. Acad Med. 2017;92(6):736–8. *This manuscript was included since Pr Ten Cate is a leading thinking in the field of entrustment decisions and we wanted to include his point of view and references as a starting point for our exploration of the topic.


**Figure 1:**
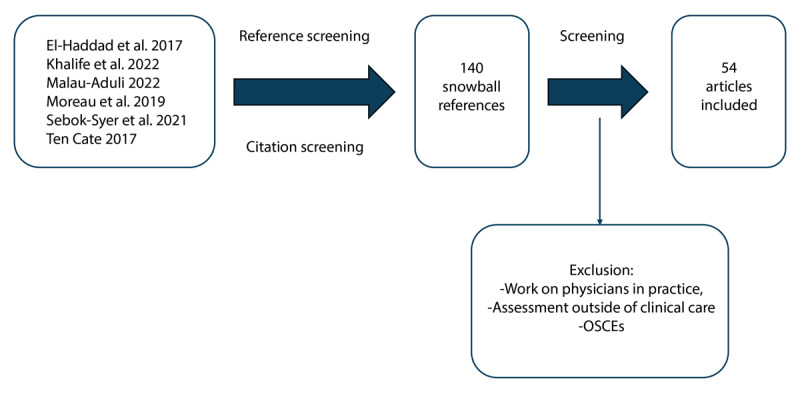
Manuscript screening.

The principal author read titles and abstracts for all 140 snowball references to determine inclusion or exclusion. She would seek input from other team members when in doubt. To be included in our archive, papers had to tackle the topic of patients assessing medical trainees at the point-of-care. We excluded work focused on physicians in practice (i.e., not trainees) and assessments done outside of clinical care, such as objective structured clinical examinations (OSCEs). At the end of the screening, 54 manuscripts were included in the archive and thus in the analysis (Figure 1).

### Analysis

We applied the empirical discourse methodology as proposed by Hodges et al. [16]. The first author initially coded the six key articles—as per Hodges’ critical empirical Discourse Analysis—for “conceptualization”, “ways in which the patients participate”, “what is made possible”, “challenges”, and “assessment” (which included content, development, validation, format, etc). She reviewed all excerpts and created a first organization of the data which she presented to the team members. She iteratively identified ways of integrating patients into the assessment of medical trainees by examining three types of statements: those that prescribe what one “should” or “must” do to establish validity, those that discuss the consequences and implications of such claims, and those that assert truths about patient-based assessments. The first author subsequently revised the coding structure after team discussions and continued the coding. We used MaxQDA to organize the data extraction for the 54 included articles. Data extraction was done based on alphabetical order based on the manuscript name. After coding another ten articles, the first author reviewed all excerpts and revised the coding structure/data organization. This was presented and discussed with team members. The first author then clarified and revised the coding structure. This back-and-forth process was used as one way to mitigate a potential confirmation bias. For example, the first author initially identified a discourse focused on the validity of patient-based assessment. Team members questioned the true prevalence of that discourse in the data versus a potential over interpretation of the principal author’s focus on validity in her program of research. Excerpts were revisited in their initial context, and in relation to the other data extracted from the manuscript. Through team discussion, we determined that discourse was not as present as initially inferred by the principal author and as such, it was put aside. The first author coded the remaining articles and created an initial/preliminary result table to be discussed in a team meeting. After coding and analysing data from 54 articles we reached a point of data sufficiency/information power. That is, during the process of coding those articles, we did not find new information to add or nuance to what we had already identified. The team did iterative revisions of the data interpretation until a consensus was achieved.

Team members’ collective expertise and experiences informed the analysis and data interpretation. We purposefully brought together team members that have experience with assessment (CS-O, LB, MM, DS, IB), Competency-Based Medical Education (CBME) (IB, TD, CS-O, DS), and with experience in Discourse Analysis (CS-O, TD). Some team members have ‘on-the-ground’ clinical-supervisory experiences (IB, DS, MM, M-EP), while others contribute theoretical and conceptual perspectives (CS-O, TD, LB). In addition to the academic perspective, we thought it was quintessential to have a patient on the team to help us navigate and interpret the different discourses we identified in the literature. With help from our institution *Programme de simulation humaine et de participation citoyenne* [program for human simulation and citizen participation] we were able to recruit a patient-partner to join our team (ILS). Bringing together complementary expertise and experiences helped us to achieve a robust interpretation of our data/archive.

## Results

### Discourses

Three discourses were the most distinguishable: (1) patients as survey-fillers, (2) patients as feedback providers, and (3) patients as part of programmatic assessment, as summarized in Table 2. We do not consider these to be the only possible ways for patients to participate in the assessment of medical trainees. Still, these discourses were observed most strongly in our analysis. Further, we do not believe these discourses to be mutually exclusive, given that we witnessed instances where more than one discourse was present in a given publication. In the first discourse, patients as survey-fillers, the integration of patients in the assessment of medical trainees is presented as necessary to meet hospital or specialty accreditation requirements. While the accreditation requirement remains true for the other two discourses, the justification and motivation explicitly put forward is what distinguishes them from the first discourse. In the second discourse, patients as feedback providers, patients are seen as a rich source of feedback that can help medical trainees gain competence. In the third discourse, patients as part of programmatic assessment, patients are seen as active players that can contribute a missing piece in the assessment puzzle.

**Table 2:** Summary of the three discourses.


	SURVEY-FILLERS	FEEDBACK PROVIDERS	PART OF PROGRAMMATIC ASSESSMENT

**Defining characteristic**	Accreditation norm to be filled	Training towards patient-centered care	Providing a holistic perspective

**Ways in which patients participate**	Filling out satisfaction surveys	Contributors to trainee development towards competent physicians	Potentially contributing at several moments of the conceptualization, implementation, administration and evaluation of assessment

**Focus**	Number of surveys required for acceptable reliability	Structuring feedback and making it accessible for trainees	Recognition of patients’ necessary complementary expertise

**What is made possible**	Meeting accreditation requirements	A catalytic effect on trainees’ development and acquisition of communication and interpersonal skills	A more holistic view of performance/ability/care that values the patient perspective

**Challenges**	Feasibility and return on investment	Trainee receptivity	Determining what can be assessed, when and how


We observed that the collected assessment data, for example, patient satisfaction or their assessment of communication skills or professionalism, did not cluster around any specific discourse. Interestingly, across all three discourses, there was a mix of both satisfaction and evaluation data related to “soft skills” like communication skills. However, what truly set these discourses apart was how the data was leveraged, which we describe further below.

### Patients as Survey-Fillers

In this discourse, patients are primarily positioned as respondents whose surveys are used to satisfy accreditation and reliability requirements.

Medical trainees will be expected to demonstrate communication, interpersonal skills, and professionalism in their future practice. With the shift to an outcomes-based approach to education, that is, Competency Based Medical Education (CBME), accreditation bodies thus expect programs to attest that these skills are mastered by their trainees. Consequently, some accreditation bodies explicitly state that patients should contribute to the assessment of these skills. However, programs are responsible for implementing patient-based assessments, using the resources available to them.

“***Patient surveys** are recommended by the ACGME [Accreditation Council for Graduate Medical Education] **as most appropriate** at measuring the three process-oriented competencies—namely, patient care, interpersonal and communication skills, and professionalism*” [19].

In this discourse, patients are asked to fill out forms. The integration of patients in the assessment of medical trainees seems to be mainly driven by accreditation standards. Fulfilling accreditation bodies’ requirements was documented as a significant and dominant reason for involving patients in the assessment of medical trainees (e.g., Ju et al.; Joshi et al.; El-Haddad et al.) [20,21,22]. There was little, if any, discussion about the potential benefits to trainees and or patients. Patients seemed to be viewed primarily as individuals who completed surveys to fulfill accreditation requirements.

Given the focus on meeting an accreditation norm, the discussion was mostly around gathering psychometric evidence to support an objective integration of patients in the assessment of trainees. The concerns mainly revolved around the number of patients that needed to complete the surveys to achieve acceptable reliability (e.g., Dine et al.; Garra et al.; Mahoney et al.) [23,24,25], the tools used [19,26,27] and survey format, for example, paper versus electronic [24,28]. Unfortunately, there was a notable lack of discussion regarding how the survey data was utilized. Because of the dominant focus on the psychometric data, and the quantification of the patient surveys we could infer that the data collected was used as evidence for meeting the accreditation requirements. However, this was not always explicitly stated.

With the conversation largely focused on feasibility and return on investment concerns, there was an emphasis on achieving adequate reliability while minimizing costs. We saw a quest for the ‘best’ survey/tool (e.g., Weaver et al.) [29] as authors “*engage[d] in a systematic scale development process to obtain a psychometrically sound Communication Assessment Tool (CAT)*” [30]. Also predominant in this discourse is the endorsement of tools and toolboxes (e.g., Qu et al.; Stausmire et al.; Wood et al.) [31,32,33].

“*For evaluating interpersonal and communication skills, **the Toolbox suggests using** the 360-degree evaluation in addition to surveying patients, and conducting OSCEs with SPs*” [21].

There seems to be a quest for demonstrating that the norm was met, and that it was done as rigourously and objectively as possible. Evidence to support these claims include the ‘best’ tools being used to compare trainees’ skills pre-post intervention (e.g., Claramita and Majoor) [34] or to compare ratings from different raters (e.g., Brinkman et al.; Byrd et al.; Henkin et al.) [35,36,37]. Although it was not dominant in the data, there was sometimes an undertone that seemed to hint that patients, their caregivers and/or relatives were not as good assessors since their scores didn’t correlate highly with clinicians’ scores.

“*When we compared the association between mean ratings among pairings of the three groups, nurse and physician ratings were significantly correlated (r = 0.562, P = 0.01), but **parent mean ratings were unrelated** to attending physician (r = –0.174) and nurse (r = 0.002) mean ratings*” [36].

Challenges observed in this discourse revolved around the resources required to gather patient data, be it in the number of surveys required to achieve sufficient reliability (e.g., Dine et al.) [23] or the human resources required to set up, implement, and execute the data collection, as illustrated below:

“*Although each evaluation took only approximately 6 minutes to complete, at least 50 evaluations would be required per intern to evaluate each reliably. This replicates the method used by other studies that have been found to require at least 50 patient evaluations*” [15,17,24,25].

### Patients as Feedback Providers

In this discourse, patients are framed as sources of feedback that can support trainees’ learning and development into more competent physicians.

With the growing body of evidence linking effective communication and strong patient relationships to positive patient health-related outcomes [23,35,38,39,40,41], patient-centered care considerations are increasingly incorporated into training programs [22,42,43]. The goal is to foster the skills that contribute to the delivery of high-quality healthcare and improved patient related health outcomes through the use of patient feedback [3,44,45,46]. Consequently, the potential to increase positive health outcomes is put forward as a main motivation or justification for the integration of patients in the assessment of medical trainees.

In the patient as feedback providers, it is believed that trainees can develop the necessary skills for strong patient-physician relationships through patient feedback [3]. Within this discourse, the emphasis is thus on helping trainees to build relationship skills, facilitating trainee learning, and promoting their development into competent physicians [47].

“*Specifically, residents generally agreed strongly that patient feedback is important to their professional development and that patient communication is important for providing quality patient care. They also agreed that patient feedback increases their confidence in their communication skills and changes the way they communicate with future patients*” [48].

In the discourse based on a patient as a feedback provider, the integration of patients—in the assessment of medical trainees—is mainly focused on providing feedback. The feedback provided was sometimes about communication and interpersonal skills [13,48,49]. It could also be based on patient satisfaction surveys [37,41]. The assessment data that patients provided were thus “*collected and summarized to provide residents with individual feedback on their competencies*” [12]. In other words, the assessment data was not used to make decisions about trainee progression in a program, in most contexts.

“*The patient feedback would not be used to assess the communication skills of GPTs [General Practice Trainees], but to provide the GPTs more or better insight into their (patient-centered) consultation skills*” [13].

Should there be a need or desire to use patient assessment data for other purposes than providing feedback, more evidence would be required to support such uses.

“*If, however, patient evaluations contributed to student feedback and monitoring rather than formal assessment, the need for such high levels of reliability might be less compelling*” [49].

The focus of this discourse is thus on the catalytic effect of patient feedback on trainees’ development and acquisition of communication and interpersonal skills (with the underlying objective of improving patient satisfaction and health outcomes). Indeed, one thing highlighted in this discourse is the growth/development of trainees.

“*One study of internal medicine residents demonstrated significant improvement among low-performing residents in response to structured feedback on patient satisfaction ratings*” [14].

Given that the main focus is integrating patients to provide helpful feedback to medical trainees, there is significant consideration given to the importance of structuring that feedback, namely via feedback facilitators—often a faculty member [2,14,33,38,48,50].

One challenge with patients as feedback providers could be residents’ receptivity. While most trainees might be receptive to patient feedback, some seemed to be resistant or hesitant when considering patient feedback. This varied from a complete disregard for patient feedback (e.g., Bogetz et al.; Castonguay et al.) [12,51] to a consideration for some feedback, mostly aimed at communication or interpersonal skills (e.g., Thomas and Hellman) [52]. Feedback was mostly discarded when “*it did not align with their self-perceptions*” [51]. One way to increase trainee receptivity to patient feedback was by using feedback facilitators (e.g., Bogetz et al.; Brinkman et al.; Cope et al.; Khalife et al.; Rassbach et al.; Wood et al.) [2,14,33,38,48,50]. This provided trainees the opportunity to interpret patient feedback with guidance from the facilitator, thus increasing their receptivity and the catalytic effect of the feedback.

### Patients as Part of Programmatic Assessment

In this discourse, patients are treated as complementary stakeholders whose perspectives contribute to a broader, more holistic programmatic assessment of trainee performance.

Supervisors cannot observe all of their trainees’ encounters with patients and do not always have the best vantage point (e.g., Byrd et al.; Dijk et al.) [36,47]. Some skills or attitudes, such as communication skills, are best assessed by those with first-hand experience with the trainees [47]. Some argue patients, or their near ones, are best situated to provide accurate information about the student-patient relationship.

“*The patient’s perspective of residents’ communication skills is important given the fundamental role of patient-centeredness in high-quality care. Patients’ evaluations provide a different perspective of residents’ behavior, empower patients to contribute to medical education, and give insight into improving the patient-physician interaction*” [7,8,9,10,23].

The integration of patients in the assessment of medical trainees is also justified from a programmatic assessment perspective [2,3]. Many authors purport that patients’ points of view complete supervisors’ observations, thus providing a more holistic assessment of trainees. As such, there is an emphasis on the complementarity of patients’ perspectives to other points of view (versus a comparison as seen in the previous discourse) (e.g., Bogetz et al.; Byrd et al.) [36,38]. Some authors argue that integrating patients into the assessment of medical trainees promotes a more authentic representation of the society in which they will practice [53].

“*By virtue of being intimately coupled to performances of residents’ interpersonal skills and behaviours, patients hold the potential to augment traditional assessment approaches and shed light on less represented competencies such as communication, advocacy and professionalism* [6,54], *thus provide a more holistic picture of learners’ competence*” [2,55].

Lastly, there is a call for considering the integration of patients into the assessment of trainees as part of a validity argumentation.

“*In workplace-based curricula that incorporate EPAs [Entrustable Professional Activities], another dimension is added. The question is not only whether the learner performed well but also whether the learner is ready for a decrease in the level of supervision. This new dimension is both an assessment issue and a patient safety issue. Is the learner ready to be left alone with the patient? The patient then becomes part of the validity argument*” [56].

Using patient assessment data, in this discourse can go beyond providing feedback to medical trainees or satisfaction data to programs. In this discourse, patient assessment data can be used to identify medical trainees who need remediation, and it can also be used to predict future issues in performance [37,49].

*However, in analyzing the patients’ responses it is obvious that ‘clear explanations’ and ‘frequent visits to the patients’ by the student are both highly associated with the patient wish for the choice of his/her student as future doctor. These findings are consistent with previous findings which correlate patient satisfaction with physician affective behavior* [3,4]. *The only behavior which stood out in its lack of correlation with patient choice was ‘family contact’, perhaps indicating that patients do not consider important the student’s contact with their families. Alternatively, patients may be unaware of the extent of the students’ contacts with their families* [37].

In some instances, patients participate in structured and summative assessments [47]. Furthermore, feedback provided by patients is perceived, by some, as a form of competence assessment [38].

“*One facet of patient centeredness is patient experience; to assess this the IOM [Institute of Medicine] advocates the use of patient feedback. Patient feedback is an important tool to assess the competency of residents*” [38].

“*An EPA developed after consulting with patients (as well as health care providers) could therefore represent both patient and clinician expectations of trainees; such an EPA could direct these physicians-in-training toward providing more patient-centered care*” [22].

In this discourse, patients are seen as individuals that can contribute to the identification of content and skills that could be assessed [22,57]. As such, they can contribute to the assessment development and go beyond filling out surveys [57].

“*We can also incorporate patients in the process of identifying and developing outcome measures that reflect aspects most important to them*” [57].

## Discussion

The purpose of this paper was to gain a deeper understanding of the current discourses surrounding the integration of real patients in the assessment of medical trainees. We opted for an empirical Discourse Analysis to gain a deeper understanding of the current discourses surrounding the integration of real patients in the assessment of medical trainees. Our data showed that predominant patient-based assessment practices remain tethered to traditional hierarchies and accreditation-driven metrics that limit patient voices, as evidenced by their frequent presence in the data archive. Shifting discourses to recognize patients as active contributors can catalyze broader systemic changes, promoting inclusivity, accountability, and reflective practice in medical education [57]. This transformation would align with calls to embed equity, social accountability, and health systems responsive to patient and community needs into medical education.

More specifically, our findings show a continuum of integration of patients in the assessment of medical trainees, from survey-fillers (superficial integration) to recognized stakeholders as part of a programmatic assessment. Unsurprisingly, these discourses align with Nemir et al.’s [1] scoping review results, confirming the major struggles but also opportunities when integrating real patients in the assessment of medical trainees. Unfortunately, while there is an increasing effort to integrate patients into medical education, we still have work to do to see more real patients in the assessment of medical trainees [1,58].

The first discourse, patients as survey-fillers, in particular, seemed like little thought/effort led to it. We base this observation on the little evidence we found regarding an explicit exploration of the role/place patients should/could have in assessing medical trainees. There was little discussion in the articles that aligned with this discourse about the potential benefits to trainees and patients. Patients appeared to be viewed primarily as sources of survey data used to demonstrate compliance with accreditation requirements [2]. The emphasis on psychometric measures and quantifiable outcomes suggests that these assessments functioned less as tools for learning and more as evidence to satisfy such standards [2]. A major concern in this discourse was the resources required to sustain these processes.

In the second discourse, patients as feedback providers, we observed a more structured or thought-out approach to integrating patients in the assessment of trainees, stating reasons such as improving and ensuring good care through better training. A caveat in this discourse is that not all trainees were as receptive to patient feedback [59]. Working with mentors or coach helped residents integrate patient feedback. Khalife et al. [2] also reported that not all trainees are open to feedback in the same way, but working with a mentor who acts as a translator helps to make the patient feedback more accessible to trainees.

In the third discourse, patients as part of the programmatic assessment, there was a more structured and prominent place for patients’ voices. The integration of patients’ voices in this discourse was threefold: 1- as assessors, 2- as informing what could/should be assessed, and 3- as a driver for assessment (i.e., high-quality patient care is an underlying motivation for quality assessment practices) which was often referred to in the context of entrustment [56]. This discourse aligns with current recommendation of patient-centered research, where patients are at the center of the process, and not just an outcome measure [54]. In other words, patients weren’t seen as accessory to the assessment process, they were seen as an intrinsic and complementary part of the assessment mosaic [60,61].

One overall challenge to integrating patients into the assessment of medical trainees is determining what they can assess, when, and how [2,13]. This challenge seem to shape discourse and seems a common limitation to the integration of patients in the assessment of trainees [1,2]. Some authors questioned if the patient-doctor relationship would prevent patients from being honest and/or objective in their assessments of medical trainees for fear of hindering the relationship [1,33,37]. Different factors, such as age, and past relationship with the health care provider, come into play. In addition, the credibility of the patients and the legitimacy of their perspective on matters related to assessment can sometimes be called into question (e.g., Bogetz et al., Reinders et al.) [13,51]. These challenges highlight the importance of integrating patients early on and having conversations with them to determine what, when and how they can contribute to assessing medical trainees as highlighted in the third discourse [1,6].

Although we didn’t approach this Discourse Analysis from a Foucauldian perspective, we would be remiss if we didn’t highlight the power relations [62] that transpire regarding the integration of patients in the assessment of medical trainees that we could observe using an empirical Discourse Analysis [16,55,63]. Similar power dynamics and struggles were also observed by Selbach et al. [62]. In the first discourse of patients as survey-fillers, there is an undertone of what the patient can do for the program or hospital (meeting accreditation requirements). In the second discourse, patients as feedback providers, the undertone is about what patients can do for trainees by contributing to their training and their development into competent physicians. Subsequently, there would be a return on investment for all patients that would benefit from care provided by competent physicians attuned to patients’ needs. Lastly, in the patients as stakeholder discourse, the undertone seems to be about patients being a part of the system that shapes future physicians by adopting different roles and responsibilities (e.g., identify medical trainees with remedial needs) in different contexts. Nemir et al. [1] highlight the need for a structured, concerted effort to integrate real patients into the training of future health professionals. This has been emphasized by different authors, namely in the Montreal Model of Patient Engagement [64], Sebok-Syer et al. [57] and Ten Cate [56]. Likely, a concerted effort could contribute to better power distribution among all stakeholders, and engage patients’ voice in the assessment of medical trainees. In addition, the shift in power must come from within the programs to see concrete changes, as suggested by Molloy and Bearman [65] and van de Walle et al. [66].

Limitations of this work include our focus on the scientific literature on patient-based assessment. While this field is expanding rapidly, our archive was limited to published literature, which emphasizes discourses vetted through peer review but may exclude less conventional perspectives from practitioners or laypeople that could influence assessment practices. This approach to Discourse Analysis may smooth over local innovations and initiatives, to represent only what has made it through the peer review process. Then again, many local initiatives are informed by the scientific literature. Discourse Analysis is inherently shaped by the theoretical stance and reflexive positioning of the research team. To avoid having only one perspective, our analysis was shaped by the team’s diverse backgrounds in measurement, cognitive psychology, and assessment. In addition, to incorporate a broader viewpoint (not limited to academia), our research team included a patient-partner who actively contributed to interpreting the analysis, challenging our assumptions and underlying conceptions. Finally, the third discourse, patients as part of the programmatic assessment, is supported by fewer empirical studies than the other two. Further research is needed to better understand this discourse and its potential impact on the development and monitoring of patient-based assessment.

## Conclusion

Because assessment is widely recognized as a powerful driver of learning—and learning medicine fundamentally entails learning to care for patients—it follows that patients should play a central role in assessment. Accordingly, there have been calls for their involvement at every stage, from designing assessment tools to contributing to final evaluation decisions [56,57]. Discourse Analysis provided a critical lens for examining discourses underlying the integration of real patients in the assessment of medical trainees. By illuminating these discourses, this study deepens our understanding of why patient involvement takes the forms it does and suggests that moving toward positioning patients as part of programmatic assessment model may offer a more meaningful basis for patient partnership in the assessment of medical trainees.

## Other disclosures

The first author used Perplexity.ai to revise sentences to improve text clarity and fluidity. The first author critically revised all of Perplexity.ai’s suggestions and adapted them before integrating them into the text.

## Previous presentations

Findings from this study have been presented at the Forum international francophone de pédagogie des sciences de la santé, Montréal, Québec, Canada, in June 2023 and at the International Conference on Academic Medicine in Québec City, Québec, Canada, in April 2023.
